# Immunohistochemical Evaluation of Acetylcholinesterase-Positive Neurons in the Brain Cortex of Rats After Administration of Rebaudioside A

**DOI:** 10.3390/brainsci15080845

**Published:** 2025-08-08

**Authors:** Karol Rycerz, Krzysztof Balawender, Tommaso Cassano, Agnieszka Żuryń, Marcin B. Arciszewski, Jerzy Walocha, Agata Wawrzyniak

**Affiliations:** 1 Department of Animal Anatomy and Histology , Faculty of Veterinary Medicine, University of Life Sciences, 20-033 Lublin, Poland; marcin.arciszewski@up.lublin.pl; 2 Department of Normal and Clinical Anatomy, Medical College of Rzeszow University, 35-615 Rzeszów, Poland; 3 Department of Medical and Surgical Sciences, University of Foggia, 71122 Foggia, Italy; tommaso.cassano@unifg.it; 4 Department of Histology and Embryology, Nicolaus Copernicus University in Toruń, 85-067 Bydgoszcz, Poland; azuryn@cm.umk.pl; 5 Department of Anatomy, Jagiellonian University Medical College , 31-034 Kraków, Poland; j.walocha@uj.edu.pl; 6 Department of Histology and Embryology, Medical College of Rzeszow University, 35-615 Rzeszów, Poland; awawrzyniak@ur.edu.pl

**Keywords:** Stevia, steviol glycosides, nerve cells, memory, learning

## Abstract

Objectives: The aim of this study was to investigate the effect of Rebaudioside A (RebA) on acetylcholinesterase (AChE) immunoreactivity in cortical neurons of the rat brain. RebA is a steviol glycoside commonly used in the production of sweeteners. Beyond its application as a food additive for diabetes management, steviol glycosides have been shown to influence memory and learning processes. Methods: RebA was administered to rats at two concentrations (1 mg/mL and 2 mg/mL of water) over both short-term (15 days) and long-term (45 days) periods. Indirect immunohistochemical peroxidase–antiperoxidase staining was performed on histological frontal sections of the brain cortex. Results: Acetylcholinesterase-positive neurons were analyzed both morphologically and morphometrically. The results of the experiment revealed no significant morphological changes in AChE-immunopositive neurons, indicating an absence of neurotoxic effects associated with the sweetener in these neurons. However, the analysis demonstrated a reduction in AChE immunoreactivity, particularly after 45 days of treatment. Conclusions: These preliminary findings demonstrates that RebA affects the immunoreactivity of neurons positive for AChE. Given the observed effects, further studies should be implemented to investigate the exact influence of this dietary supplement on the cholinergic nervous system.

## 1. Introduction

Rebaudioside A (RebA) is a diterpenoid glycoside isolated from the leaves of Stevia rebaudiana Bertoni and is widely used as a high-intensity, non-caloric sweetener. It is approximately 200–300 times sweeter than sucrose, making it a popular sugar substitute in the food and pharmaceutical industries [1]. Upon oral ingestion, steviol glycosides, including RebA, undergo microbial hydrolysis in the gastrointestinal tract, yielding steviol. This metabolite is subsequently absorbed into the bloodstream, transported to the liver, and conjugated to steviol glucuronide—the principal systemic metabolite in both humans and rodents [2,3,4,5,6].

Emerging evidence suggests that steviol glycosides may exert neuromodulatory effects. In rodent models, oral administration of stevioside—which is structurally and metabolically analogous to RebA—has been shown to enhance cognitive performance and attenuate memory impairments induced by scopolamine, a muscarinic antagonist used to model Alzheimer’s-like cognitive deficits [7]. Evidence supporting the cognitive benefits of steviol-based compounds remains limited but promising. For instance, some authors have demonstrated improved spatial memory in rats following oral administration of Stevia rebaudiana extract. This effect was accompanied by decreased AChE levels in brain homogenates, likely related to the plant’s antioxidant and anti-inflammatory properties [7,8]. However, it should be noted that crude Stevia extracts and commercially available sweeteners contain other compounds. Stevia extracts contain not only various glycosides but also phytoconstituents, such as flavonoids, phenolic acids, fatty acids, proteins, and vitamins [9]. These substances may influence the results of studies obtained by other authors. In our study, pure laboratory-isolated RebA was used. Furthermore, several natural and artificial sweeteners have been investigated for their potential roles in the prevention and management of type 2 diabetes mellitus (T2DM)—a recognized risk factor for age-related cognitive decline and Alzheimer’s disease [10,11,12].

Acetylcholinesterase (AChE) is a key regulatory enzyme in cholinergic neurotransmission, responsible for the rapid hydrolysis of acetylcholine (ACh) in the synaptic cleft. AChE plays a crucial role in controlling the temporal dynamics of cholinergic signaling by modulating ACh availability. Pharmacological inhibition of AChE remains a principal therapeutic strategy in Alzheimer’s disease, aiming to prolong synaptic ACh activity and enhance cholinergic function [13,14].

Cortical cholinergic innervation in mammals arises predominantly from the basal forebrain, particularly from magnocellular neurons of the nucleus basalis of Meynert and the substantia innominata. These projections are critical for higher cognitive functions such as attention, learning, and memory, primarily mediated via muscarinic and nicotinic receptors [15,16,17,18]. In the medial prefrontal cortex (mPFC), cholinergic signaling enhances attentional performance and regulates synaptic plasticity. Additionally, cholinergic pathways influence neuroinflammatory processes and modulate gene expression involved in cognition and neurodegeneration.

The cerebral cortex is organized into six cytoarchitectonic layers, namely molecular (I), external granular (II), external pyramidal (III), internal granular (IV), internal pyramidal (V), and polymorphic or multiform (VI) [19,20]. AChE-immunoreactive (AChE-IR) neurons are particularly abundant in layers III and V, reflecting regions of dense cholinergic modulation [21,22]. At the cellular level, AChE is localized in neuronal perikarya, plasma membranes, axons, dendrites, and synaptic terminals. It is expressed in both cholinergic and non-cholinergic neuronal populations [23].

In our previous studies, we demonstrated the influence of RebA on AChE-IR neurons in the hippocampus and striatum of rats treated with the studied sweetener. These findings showed an increase in the density of the studied nervous cells after 45 days of sweetener administration in doses: 1 mg RebA/mL and 2 mg RebA/mL. A decrease in the reaction intensity of AChE-positive neurons was also demonstrated in the hippocampal CA1 field and in GP [24]. However, we observed that the number of AChE-IR cells did not change after 15 days of RebA administration at either dose in the hippocampal CA1 region and the striatum. In contrast, the immunostaining intensity decreased in these areas. [25]. The immunoreactivity of M1 muscarinic receptors was also assessed in the striatum and hippocampus. The results were different from those for AChE-positive neurons. After 45 days of administration of the tested sweetener at both doses, the number of cells that were immunopositive for M1 muscarinic receptors increased. Additionally, stronger immunostaining of neurons positive for these receptors was observed [24]. The cholinergic system includes neurons located in the basal forebrain, providing long projections to the hippocampus and cortex, which has an influence on cognitive functions [21]. The effect of RebA on AChE-IR neurons at different doses and different times of administration has not been studied so far.

Given the increasing interest in the neurocognitive effects of dietary sweeteners, particularly steviol glycosides, the present preliminary study aims to evaluate the impact of RebA on AChE immunoreactivity in the cerebral cortex of adult rats. By examining the laminar distribution and intensity of AChE-IR neurons across cortical layers, this investigation seeks to elucidate potential interactions between RebA and central cholinergic neurotransmission, thereby contributing to our understanding of its neuromodulatory potential.

## 2. Materials and Methods

### 2.1. Animals and Experimental Design

Thirty 55-day-old male Wistar rats (initial body weight: 250–290 g) were used in this study. All animals were housed individually in standard plastic cages under controlled environmental conditions (22 ± 2 °C, 12 h light/dark cycle) with ad libitum access to standard chow and water. Experimental procedures were approved by the Second Local Ethical Committee (Approval No. 22/2013), in accordance with Directive 2010/63/EU on the protection of animals used for scientific purposes. In this study, female rats were excluded according to the possible influence of sex hormones on the cerebral cortex cholinergic system [26]. Following a 7-day acclimatization period, animals were randomly allocated into two main groups based on treatment duration: Group A (*n* = 15), treated for 15 days, and Group B (*n* = 15), treated for 45 days. Each group was subdivided into three experimental subgroups (*n* = 5 per subgroup): Subgroup A1/B1 received Rebaudioside A (RebA, Sigma Aldrich, St. Louis, MO, USA) dissolved in drinking water at a concentration of 1 mg/mL (estimated dose: 65 mg/kg body weight/day). Subgroup A2/B2 received RebA at a concentration of 2 mg/mL (estimated dose: 119 mg/kg body weight/day). Control subgroups AK/BK: received only tap water. RebA solution (25 mL—total amount of daily solution) was administered daily in separate drinking bottles throughout the experimental period. All rats were kept individually in cages, and the amount of sweetener consumed was checked daily to determine the dose of RebA taken. The selection of two doses of RebA and two administration durations was informed by previous research indicating that only the highest of three tested doses produced behavioral improvements in memory [7,8]. The lower doses did not yield the desired effects. However, we aimed to investigate whether these lower doses were truly ineffective or whether their efficacy might depend on the duration of administration. In the referenced study, RebA was administered orally for 16 days. Based on this, and to assess the potential influence of prolonged exposure, we included an extended administration period in our design. The same experimental model was previously employed in our studies focusing on the hippocampus and striatum [24,25]. At the end of the treatment, animals were euthanized after the administration of ketamine and xylazine (ip, 300 mg/kg b.w. and 30 mg/kg b.w., respectively). Brains were immediately dissected and immersion-fixed in 10% buffered formalin for 12 h. Following fixation, the tissues were processed using standard paraffin embedding procedures and sectioned coronally at a thickness of 8 µm. The coordinates of the cortex were selected according to the stereotaxic atlas (A 4230 µm–A 3750 µm), which corresponds to the region between A +4.23 mm and A +3.75 mm relative to bregma [27]. Cortical layers II–VI were identified based on their cytoarchitecture. Layer II: dense population of small pyramidal and stellate cells. Layer III: moderately dense population of small-to-medium-sized pyramidal neurons. Layer IV: densely packed granule (stellate) cells forming a clearly demarcated band. Layer V: large pyramidal neurons with prominent soma. Layer VI: heterogeneous cell population with loosely packed, polymorphic neurons [28].

### 2.2. Immunohistochemistry

Immunohistochemical detection of AChE was performed using the indirect peroxidase–antiperoxidase (PAP) method. Briefly, paraffin sections were deparaffinized and rehydrated through graded series of ethanol. Endogenous peroxidase activity was quenched using 3% hydrogen peroxide. Non-specific binding was blocked with normal goat serum. Antigen retrieval was carried out in citrate buffer (pH 6.0) by microwave heating (800 W, 3 cycles × 5 min). Sections were incubated for 48 h at 4 °C with monoclonal mouse anti-acetylcholinesterase antibody (1:1000; Sigma Aldrich), followed by incubation with goat anti-mouse IgG conjugated to horseradish peroxidase for 1 h in room temperature (1:150; Sigma Aldrich). The reaction was visualized using 3,3′-diaminobenzidine (DAB; Fluka, Buchs, Switzerland) as chromogen. Counterstaining was performed with Mayer’s hematoxylin. Slides were dehydrated, cleared in xylene, and mounted with DPX mounting medium. Negative controls were performed by omitting the primary antibody and replacing it with normal goat serum. Positive controls were carried out according to the manufacturer’s recommendations. Images were captured using an Axiolab RE microscope equipped with an Axiocam 105 digital camera (Carl Zeiss, Jena, Germany).

### 2.3. Qualitative Histological Evaluation

AChE-IR neurons were identified based on cytoplasmic DAB signals and typical neuronal morphology. Their distribution was assessed in layers II–VI of the cerebral cortex. Semi-quantitative analysis of cell density was performed using the following standardized scale: (−/+) few, (+) moderately numerous, (++) numerous, (+++) very numerous. This approach was adapted based on previously published semi-quantitative methodologies used in neuropathological assessments [29,30]. Cell density was estimated in representative microscopic fields of defined cortical areas under consistent magnification (20× objective), with at least 10 fields analyzed per animal to minimize variability. The assessments were performed by two independent investigators in blinded fashion (A.W. and K.B.), and the scale was used consistently across all cases to allow for comparative evaluation between experimental groups.

### 2.4. Morphomeric Analysis

Quantitative evaluation of immunostaining intensity was conducted using the ZEN 2.3 image analysis software (Carl Zeiss). For each animal, randomly selected neurons from layers II–VI were analyzed. The staining intensity was measured in the neuronal soma in a region of interest of 1 mm^2^ in 50 cells (10 cells per animal) with brown reaction product. The intensity of free space was measured to standardize the differences in light exposure. Moreover, the results were inverted so that higher values represented darker staining and lower values represented brighter colors. Hence, immunostaining intensity expressed as optical density/mm^2^ (OD) was counted as OD = 255 − (255x/y), where x is the measured intensity in 1 mm^2^ of the cell cytoplasm and y is the measured intensity of free space [31]. All measurements were performed in blinded fashion (A.W., K.B.). Layer I was excluded due to the sparse occurrence of AChE-IR neurons.

### 2.5. Statistical Analysis

Data were expressed as mean ± standard deviation (SD). Two-way analysis of variance (ANOVA) was performed separately for each layer (Layer II–VI) to assess the effect of two factors, dose and time, on the intensity of immunostaining in AChE-IR neurons. The analysis revealed significant results of both main effects and their interactions in all analyzed layers. Multiple comparisons were performed using Tukey’s HSD post hoc test. The normality of the distribution was assessed using the Shapiro–Wilk test, and homogeneity of variance was assessed with Levene’s test. When the assumptions of ANOVA were not met, the non-parametric Kruskal–Wallis test was applied. Statistical significance was set at *p* < 0.05. Analyses were conducted using Statistica 13.3 software (TIBCO Software Inc., 2017; Palo Alto, CA, USA).

## 3. Results

**Figure 1:**
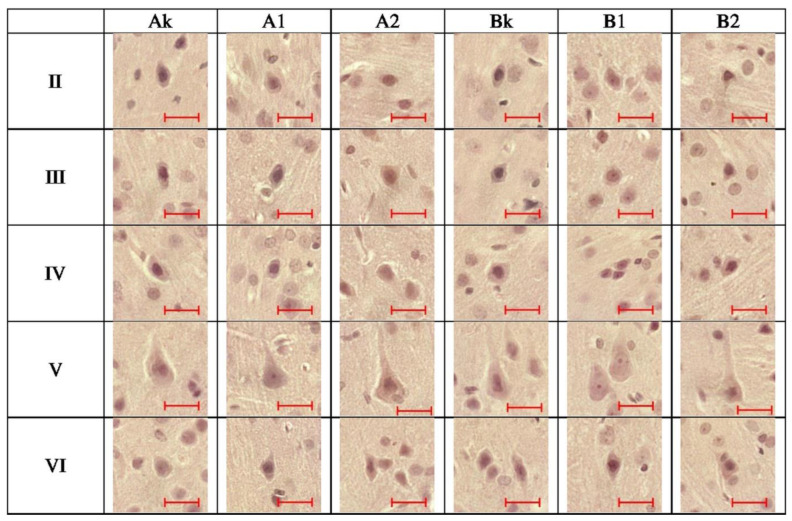
Acetylcholinesterase immunoreactive neurons from (**II**–**VI**) layers of rat brain cortex from A_K_, A_1_, A_2_, B_K_, B_1_ and B_2_ subgroups. Scale bar: 20 μm. The objective magnification used is 40×, and all images from all animals were obtained under identical conditions; the images shown are representative examples selected from randomly chosen animals.

AChE-IR neurons were identified in all cortical layers (I–VI) in the control animals and in both experimental groups (A and B) treated with RebA. The immunoreactivity was localized diffusely within the cytoplasm of the neuronal somata, with no signal detected in the nuclei. In layer II (external granular layer), numerous (+++) small pyramidal or oval AChE-IR neurons were present in the control rats and in all RebA-treated animals, regardless of dose or treatment duration. These neurons exhibited centrally located, oval nuclei. However, qualitative assessment revealed a visibly reduced staining intensity in the A1, A2, B1, and B2 subgroups compared to the controls (Figure 1(II)).

**Figure 2:**
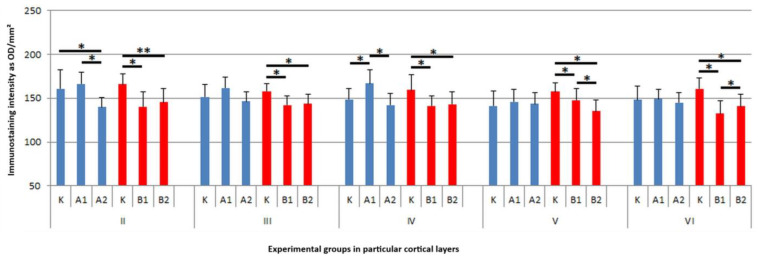
Average immunostaining intensity of acetylcholinesterase immunoreactive neurons from (**II**–**VI**) brain cortex layers in rats of AK, A1, A2, BK, B1, and B2 subgroups with standard deviation. Blue bars represent 15-day treatment and red bars represent 45-day treatment. * differences identified by post hoc Tukey HSD test considered statistically significant at *p* < 0.05; ** differences identified by Kruskal–Wallis test considered statistically significant at *p* < 0.05.

**Figure 3:**
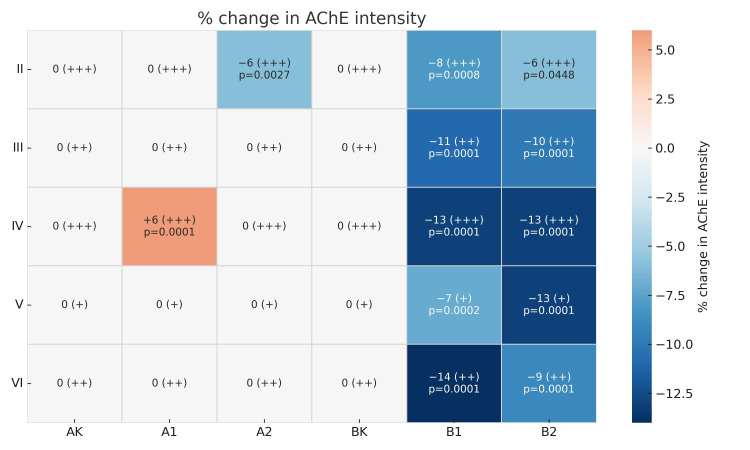
The summary heatmap presents percentage changes in AChE-IR cell intensity in neurons across cortical layers (**II**–**VI**) in rats treated with RebA for 15 or 45 days in comparison to the respective control groups (AK for 15-day treatment; BK for 45-day treatment). Each cell in the heatmap contains the percent change value (Δ%) in AChE intensity in comparison to the control group, followed by a semi-quantitative estimation of the number of AChE-IR neurons in parentheses (+ for moderately numerous cells; ++ for numerous cells; +++ for very numerous cells) and, when statistically significant, the *p*-value, indicating the difference compared to the control group. The color gradient in the heatmap reflects the direction and magnitude of immunoreactivity changes: blue tones indicate decreased intensity, red tones indicate increased intensity, and white corresponds to no statistically detectable change. Only statistically significant comparisons (*p* < 0.05) are annotated with exact *p*-values.

Moreover, in this layer, two-way ANOVA revealed significant main effects of dose (F (2,114) = 15.59; *p* = 0.000001; η^2^_p_ = 0.214772) and time (F (1,114) = 8.33; *p* = 0.004659; η^2^_p_ = 0.068116), as well as a significant interaction (F (2,114) = 20.18; *p* = 0.000000; η^2^_p_ = 0.261467). Post hoc Tukey’s test showed that rats treated with the high dose for 15 days exhibited a 6% reduction in AChE immunoreactivity compared to controls (*p* = 0.002745). While in animals treated for 45 days, there was both a reduction in the immunoreactivity of the studied protein after lower (approx. 8%, *p* = 0.0008) and higher doses of RebA (approx. 6%, *p* = 0.0448) (Figure 2(II) and Figure 3). In layer III (external pyramidal layer), AChE-IR neurons were consistently observed in moderate numbers (++), displaying typical pyramidal or oval morphology with prominent central nuclei. Weaker immunoreactivity was noted primarily in rats exposed to RebA for 45 days, especially in the A2 subgroup (Figure 1(III)). Similarly to layer II, in layer III there was a significant main effect of dose (F (2,114) = 24.69; *p* = 0.000000; η^2^_p_ = 0.302239) and time (F (1,114) = 6.72, *p* = 0.010759; η^2^_p_ = 0.055697). There was also a significant interaction between these variables (F (2,114) = 23.47; *p* = 0.000000; η^2^_p_ = 0.291686). The multiple comparisons of averages showed a decrease in reaction intensity after only 45 days of RebA administration. In group B1, this was an approx. 11% reduction and in group B2 this was an approx. 10% reduction in comparison to the control group (*p* = 0.0001, *p* = 0.0001, respectively) (Figure 2(III) and Figure 3). In layer IV (internal granular layer), numerous (+++) AChE-IR neurons were evident in all groups, characterized by oval somata and centrally positioned large nuclei. No obvious differences in staining intensity were observed between the control and experimental animals at the qualitative level (Figure 1(IV)). However, a two-way ANOVA indicated a significant main effect of dose (F (2,114) = 33.41; *p* = 0.000000; η^2^_p_ = 0.369507), time (F (1,114) = 9.09, *p* = 0.003171; η^2^_p_ = 0.073835) and a particularly strong effect of the dose × time interaction (F (2,114) = 68.70, *p* = 0.000000; η^2^_p_ = 0.546528). In layer IV, animals treated with RebA for 45 days exhibited a general approx. 13% reduction in AChE immunoreactivity for both doses (BK vs. B1, *p* = 0.0001; BK vs. B2, *p* = 0.0001). Interestingly, a subgroup-specific increase in staining intensity was noted in the A1 subgroup relative to the AK (*p* = 0.0001) and A2 subgroups (*p* = 0.0001) (Figure 2(IV) and Figure 3).

In layer V (internal pyramidal layer), moderately numerous (+) large pyramidal neurons and smaller oval AChE-IR neurons were detected. Both cell types exhibited large, round, or oval nuclei. A reduction in AChE immunoreactivity was detected in animals treated with RebA for 45 days, particularly in the high-dose subgroup (Figure 1(V)). Also in layer V, significant results for dose (F (2,114) = 19.91; *p* = 0.000000, η^2^_p_ = 0.258886), time (F (1,114) = 12.74, *p* = 0.000526; η^2^_p_ = 0.100490) and dose × time interaction (F (2,114) = 30.41; *p* = 0.000000; η^2^_p_ = 0.347882) were demonstrated. In layer V, the average AChE-IR intensity was lower in rats exposed to RebA for 45 days, with a more pronounced decline observed in the high-dose group (BK vs. B1, approx. 7% reduction, *p* = 0.0002; BK vs. B2, approx. 13% reduction, *p* = 0.0001) (Figure 2(V) and Figure 3). In layer VI (multiform layer), AChE-IR neurons were consistently present in all groups. These cells were numerous (++), varied in size (small to medium), and exhibited diverse morphologies—pyramidal, oval, or fusiform. The overall staining intensity was similar across the studied groups (Figure 1(VI)). Two-way ANOVA revealed significant main effects of dose (F (2,114) = 57.14, *p* = 0.000000; η^2^_p_ = 0.500611) and time (F (1,114) = 10.29, *p* = 0.001734; η^2^_p_ = 0.082810), as well as a significant interaction with a strong effect (F (2,114) = 69.06; *p* = 0.000000; η^2^_p_ = 0.547823). Post hoc Tukey’s test showed that rats treated with the high dose for 45 days (B2) exhibited a 9% reduction in AChE immunoreactivity compared to the controls (*p* = 0.0001), while the low-dose group (B1) showed a 14% reduction (*p* = 0.0001) (Figure 2(VI) and Figure 3). Some of the results are presented in a summary heatmap for better clarity (Figure 3).

## 4. Discussion

This study demonstrated the presence of AChE-IR neurons in all layers of the cerebral cortex in rats treated with RebA and in the control animals. The morphology and distribution of AChE-IR neurons were consistent across all experimental groups, with no detectable structural alterations following RebA administration, regardless of dose or treatment duration. AChE-IR neurons were sparse in the molecular layer (I); thus, it was not considered in our analyses. Layers II–VI contained primarily pyramidal, oval, or fusiform neurons with centrally located nuclei, consistent with typical cortical cytoarchitecture.

Although early studies suggested the potential cytotoxic or genotoxic effects of steviol glycosides [32,33], subsequent research has largely refuted these concerns, highlighting instead the therapeutic properties of compounds such as RebA. These include antihypertensive, antidiabetic, antioxidant, anti-inflammatory, and anticancer effects [34,35,36]. Our findings further support this safety profile, as no morphological abnormalities were observed in AChE-IR neurons after RebA exposure; however, we only assessed the neurons that were immunoreactive for the studied protein and not the other non-cholinergic neurons. Nevertheless, in previous work we reached similar conclusions in the AChE-IR neuron assessment for the hippocampus and striatum [24,25].

Our results showed that both the dose of RebA and its duration of treatment (15 vs. 45 days), as well as their interaction, significantly influenced the immunostaining intensity of AChE-IR neurons in the cerebral cortex (layers II–VI). Particularly strong dose × time interaction effects were observed in layers IV and VI, suggesting that increasing the duration of exposure may potentiate or modulate the action of RebA on cholinergic cells. This type of relationship may result from temporally varying changes in enzymatic activity, neuroplasticity, or adaptive regulatory mechanisms in response to various substances. It has previously been shown that modulation of AChE activity can be sensitive to both the dose and duration of exposure to a given substance, especially in cortical structures involved in cognitive and memory functions [37,38].

Semi-quantitative and morphometric analyses revealed significant changes in AChE immunoreactivity. Following 15 days of RebA administration, a significant decrease in AChE immunostaining was observed in neurons of the external granular layer at the higher dose (2 mg/mL), while the lower dose (1 mg/mL) produced an increase in immunoreactivity in the internal granular layer. These effects were not observed in other cortical layers. In contrast, after 45 days of RebA treatment, a consistent and statistically significant reduction in AChE immunoreactivity was evident in neurons across multiple cortical layers at both tested concentrations.

The present findings are consistent with the results of our previous studies. We have previously demonstrated a decrease in AChE-positive neuronal immunoreactivity in the hippocampal CA1 region of rats treated with Rebaudioside A (RebA) at a dose of 1 mg/mL for 15 days. A similar effect was observed in the striatum following administration of RebA at both 1 mg/mL and 2 mg/mL concentrations [25]. After 45 days of sweetener administration, we also reported a reduction in immunoreactivity in the CA1 layer of the hippocampus and in the globus pallidus of the striatum at both tested doses of RebA, using the same experimental model [24].

The observed decline in AChE immunoreactivity may reflect decreased enzyme concentration or activity, given the established correlation between antigen levels and DAB-based staining intensity [39]. A reduction in AChE levels could result in increased synaptic concentrations of acetylcholine (ACh), as AChE is the primary enzyme responsible for ACh hydrolysis. Elevated synaptic ACh may prolong cholinergic signaling, potentially influencing cognitive processes such as memory and learning [13,40]. ACh plays a key role in modulating attention, encoding memory engrams, and facilitating long-term potentiation [41].

Previous studies have shown that AChE inhibitors enhance ACh levels in memory-related brain regions, such as the hippocampus and entorhinal cortex, and may counteract the cognitive impairments induced by GABA receptor agonists and scopolamine [42,43]. Notably, stevioside administration has been reported to reverse scopolamine-induced memory deficits and oxidative stress, suggesting that steviol glycosides may influence the cholinergic system.

The use of dietary sweeteners such as RebA is particularly relevant in the context of metabolic disorders. Diabetes mellitus has been implicated in the pathogenesis of neurodegenerative diseases, including Alzheimer’s disease, through mechanisms involving insulin resistance, vascular dysfunction, and chronic inflammation [10,11,44,45,46,47]. Thus, natural sweeteners with potential neuroprotective properties may hold therapeutic promise.

Based on our findings, RebA does not exert neurotoxic effects on AChE-IR cortical neurons and may modulate AChE expression following prolonged administration. Due to these results, we may speculate that RebA could influence cholinergic signaling and potentially support cognitive function, which should be confirmed mainly with behavioral and biochemical tests.

Nevertheless, this study is limited by its reliance on immunohistochemistry, which, although informative, does not directly measure enzymatic activity or functional outcomes. Future studies should integrate behavioral testing to assess the influence of RebA on cognitive function. Moreover, biochemical assays of AChE activity and quantitative molecular analyses to clarify the effects of RebA on the cholinergic system should be carried out. Studies of other markers of the cholinergic system, such as choline acetyltransferase, the high-affinity choline transporter, and the vesicular acetylcholine transporter, as well as the assessment of muscarinic receptor immunoreactivity in the cerebral cortex, may also be beneficial in assessing the effects of the tested sweetener in the future. Additionally, the application of advanced imaging and digital morphometry may enhance data reproducibility and resolution. Although the number of animals per group was limited (n = 5), the sample size was sufficient to detect statistically significant effects using appropriate statistical methods. The group sizes were consistent with those used in previous studies employing similar experimental designs [24,25,48].

## 5. Conclusions

In conclusion, while RebA administration did not alter the morphological features of AChE-IR neurons in the rat cerebral cortex, morphometric evidence indicates a reduction in AChE immunoreactivity, particularly after long-term exposure. These preliminary findings shed light on the need for further investigation into the cognitive and neurochemical implications of RebA as a dietary compound with potential neuromodulatory effects.

## Data Availability

All data can be assessed by the first author, and supporting reported results can be obtained directly from the first author. The data are not publicly available due to ethical reasons.
